# Multiparty Quantum Key Agreement Based on Quantum Search Algorithm

**DOI:** 10.1038/srep45046

**Published:** 2017-03-23

**Authors:** Hao Cao, Wenping Ma

**Affiliations:** 1 Xidian Universitity, State Key Laboratory of Integrated Service Networks, Xi’an, 710071, China; 2 University of Science and Technology of Anhui, School of Information and Network Engineering, Chuzhou, 233001, China

## Abstract

Quantum key agreement is an important topic that the shared key must be negotiated equally by all participants, and any nontrivial subset of participants cannot fully determine the shared key. To date, the embed modes of subkey in all the previously proposed quantum key agreement protocols are based on either BB84 or entangled states. The research of the quantum key agreement protocol based on quantum search algorithms is still blank. In this paper, on the basis of investigating the properties of quantum search algorithms, we propose the first quantum key agreement protocol whose embed mode of subkey is based on a quantum search algorithm known as Grover’s algorithm. A novel example of protocols with 5 – *party* is presented. The efficiency analysis shows that our protocol is prior to existing MQKA protocols. Furthermore it is secure against both external attack and internal attacks.

Since the first quantum key distribution (QKD) protocol known as BB84^1^ was proposed by Bennett and Brassard in 1984, quantum cryptography has been attracted more and more attention, and many kinds of schemes such as QKD^2^,^3^,^4^, quantum secret sharing (QSS)^5^,^6^,^7^,^8^,^9^, quantum direct communication(QDC)^10^,^11^,^12^,^13^, quantum privacy comparison (QPC)^14^,^15^, have been proposed. Especially, QKD has received wide attention because of its numerous applications in quantum communication. Different from the classic cryptography schemes, quantum protocols that are based on the principles of quantum mechanics, could provide unconditionally security. Hence, quantum cryptography is innately superior to the classic cryptography.

Anther very important topic named Quantum key agreement(QKA)^16^,^17^,^18^,^19^,^20^,^21^,^22^,^23^,^24^,^25^,^26^,^27^,^28^,^29^ also received widespread concerns. Compared with QKD protocols in which one participant distributes a predetermined secret key to the other participants, QKA protocols require that all participants need to negotiate mutually and equally to derive a common secret key, and any nontrivial subset of participants could not fully determine the target key. Furthermore, any unauthorized users cannot extract the key through illegal means. Hence, the justice and fairness can be better reflected in the procession of QKA protocols because all participants are involved in the selection of the target key *K* and their contribution to it are equal. In 2004, the firstly QKA protocol (ZZX protocol)^16^ based on Einstein - Podolsky - Rosen (EPR) pairs was proposed by Zhou, Zeng and Xiong. However, in 2009, Tsa and Hwang^17^ pointed out that ZZX protocol is not a fair QKA because one party could fully determine the target key without being detected, and they proposed an improvement one (TH protocol)^18^. Unfortunately, TH protocol is also not a really QKA because the shared key is produced based on random measurement results without negotiation. In 2004, based on maximally entangled states, Hsueh and Chen also proposed a QKA protocol (HC protocol)^28^. In 2011, Chong, Tsai and Hwang^18^ claimed that HC protocol is susceptible to eavesdropping attack and internal attacks. In 2010, Chong and Hwang proposed the first successful QKA protocol (CH protocol)^19^ based on BB84 by using the technique of delayed measurement. In 2013, Liu, Gao, Huang and Wen proposed the first secure multiparty quantum key agreement (MQKA) protocol (LGHW protocol)^20^ by utilizing single particles. In the same year, Sun, Zhang and Wang *et al*.^29^ improved the LGHW protocol and the efficiency is improved obviously. Subsequently, several QKA and MQKA^21^,^22^,^23^,^24^,^25^,^26^,^27^ protocols were proposed.

Furthermore, quantum search algorithms (QSA)^30^ are also a research focus in quantum theory, and are famous for the Grover’s algorithm. The target could be probabilistic found in an unsorted database by executing the Grover’s algorithm which is faster than the best known classical search algorithms. Grover’s algorithm plays an important role in quantum computation and quantum communication. Recently, based on the ideas of QSA, some quantum protocols, liking QSS^6^, QPC^14^ and QDC^31^,^32^, have been proposed.

As far as I know, all existing QKA protocols are based on either BB84 or entangled states, and the QKA protocol based on QSA has not yet appeared. The research of the QKA protocol based on QSA still is blank. This study proposes a MQKA protocol based on QSA for the first time. In the proposed scheme, the idea of quantum dense coding is used. Each participant encodes his or her secret key by a unitary operation, and makes a two-particle quantum measurement to extract the common key. The security and efficiency analysis shows that our protocol is prior to existing MQKA protocols. The rest of our paper is structured as follows. Section 2 introduces some notions and properties of QSA. Section 3 describes the presented protocol in detail, the correctness of it is showed, and a novel example with 5-party protocol is presented. Section 4 analyzes the proposed scheme and compares it to other schemes. Finally, the conclusion of this paper is given in section 5.

## Results

### Preliminaries

Here we tackle some notations and properties of the Quantum Search Algorithm (QSA) with two quantum particles input. Owing to that Grover’s QSA is one of the most famous of all the QSAs, we only discuss the notations and properties of it.

The Grover’s QSA can be described as follows. Let the database be a two-particle quantum state |*S*〉 = |+ +〉, and *w* ∈ {00, 01, 10, 11} be the search target. One can perform two specific unitary operations on |*S*〉 = |+ +〉 repeatedly to find the target. Here, we firstly give some notations adopted in this article.

Let *w* ∈ {00, 01, 10, 11}, define |*S*_*w*_〉 as follows:


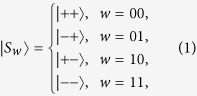


Two specific unitary operations can be described as follows.


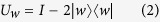



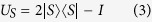


where *w* ∈ {00, 01, 10, 11} and *S* ∈ {+ +, − +, + −, − −}.

Grover’s QSA possesses two special properties as follows.

**Property 1.** Ref. 32 Let *w*_*i*_ ∈ {00, 01, 10, 11} (*i* = 1, 2, 3, 4). Then 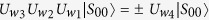 if and only if 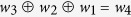.

**Property 2.** Ref. 14 Let *v, w*_1_, *w*_2_ ∈ {00, 01, 10, 11}. Then 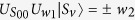 if and only if 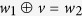.

The following **Theorem 1** and **Theorem 2** generalize the **Property 1** and **property 2** from |*S*_00_〉 to |*S*_*w*_〉 with any *w* ∈ {00, 01, 10, 11} separately.

**Theorem 1.** Let *w, w*_*i*_ ∈ {00, 01, 10, 11} (*i* = 1, 2, 3, 4), then 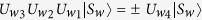 if and only if 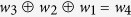. More generally, let *n* be an odd positive integer, and *w, v, w*_*i*_ ∈ {00, 01, 10, 11} (*i* = 1, 2, …, *n*), then 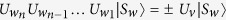 if and only if 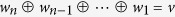.

**Proof.** (1)Firstly, we show that 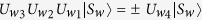 if and only if 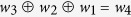.

- If 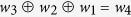 and *w*_1_ = *w*_2_, then *w*_3_ = *w*_4_, and it is obviously that 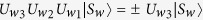. Similarly to the cases *w*_1_ = *w*_3_ and *w*_2_ = *w*_3_.
- If 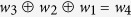, and *w*_1_, *w*_2_ and *w*_3_ are different from each other, then |*w*_1_〉, |*w*_2_〉, |*w*_3_〉 and |*w*_4_〉 are orthogonal to each other because of the relation 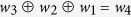. In this case, we can get
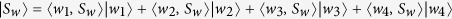
- Hence,
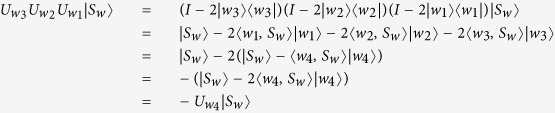
- If 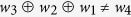, let us show that 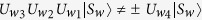.

Denote 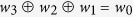. From (a) and (b), we can easily get 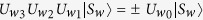. Suppose the equation 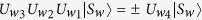 holds, then 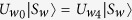 or 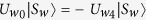.

In the former case, we have


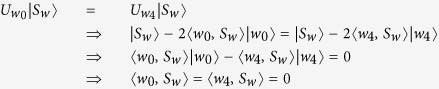


a contradiction to the fact that 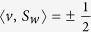 for any *v* ∈ {00, 01, 10, 11}. The same conclusion of the second case can be got similarly. Hence, 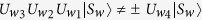.

From (a), (b) and (c), we can get 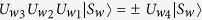 if and only if 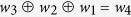.

(2) Secondly, we show that 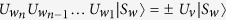 if and only if 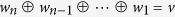. We will give the proof by using the mathematical induction to the odd positive integer *n*.

- *n* = 1, the result is trivial.
- Suppose that the result is correct in the case of *n* = *k*, where *k* is a positive odd integer. That is to say, 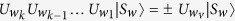 if and only if 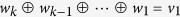,where 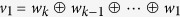. When *n* = *k* + 2, we have

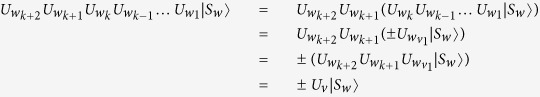


where 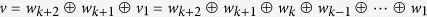.

Hence, 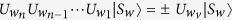 if and only if 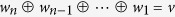.

**Theorem 2.** Let *w, v, w*_0_, *w*_1_ ∈ {00, 01, 10, 11}. Then 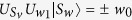 if and only if 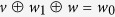.

The correctness of **Theorem 2** could be verified for each value of the tuples (*w, v, w*_0_, *w*_1_) ∈ {00, 01, 10, 11}^4^ one by one.

From **Theorem 1** and **Theorem 2**, we can get **Theorem 3** at once.

**Theorem 3.** Let *n* be an odd positive integer, and *w, v, w*_*i*_ ∈ {00, 01, 10, 11}, where *i* = 0, 1, …, *n*. Then 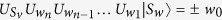 if and only if 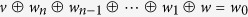.

**Theorem 4.** Let *w, w*_0_, *w*_1_, *w*_2_ ∈ {00, 01, 10, 11}. Then 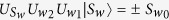 if and only if 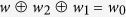. More generally, let *n* be a positive even integer, and *w, w*_*i*_ ∈ {00, 01, 10, 11} (*i* = 0, 1, …, *n*), then 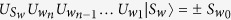 if and only if 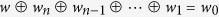.

**Proof.** (1)Firstly, we show that 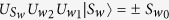 if and only if 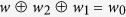.

- If *w*_1_ = *w*_2_, the result is trivial.
- If 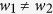,suppose {*w*_1_, *w*_2_, *w*_3_, *w*_4_} = {00, 01, 10, 11},then |*w*_1_〉, |*w*_2_〉, |*w*_3_〉 and |*w*_4_〉 are orthogonal to each other. In this case, we can get

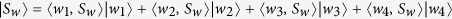


Now, we show that there exists *w*_0_ ∈ {00, 01, 10, 11} such that 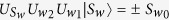.


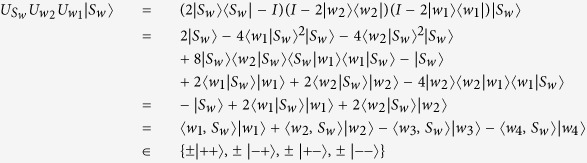


Hence, we can select a proper *w*_0_ ∈ {00, 01, 10, 11} such that 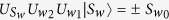, and we can easily get the relation 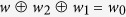 from Table 1.

(2)From (1) and Theorem 1, we can easily get the correction of the proposition that 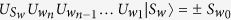 if and only if 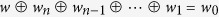, by using the mathematical induction similar to the proof of (2) in **Theorem 1**.

### The Proposed QKA Protocol

**Figure 1:**
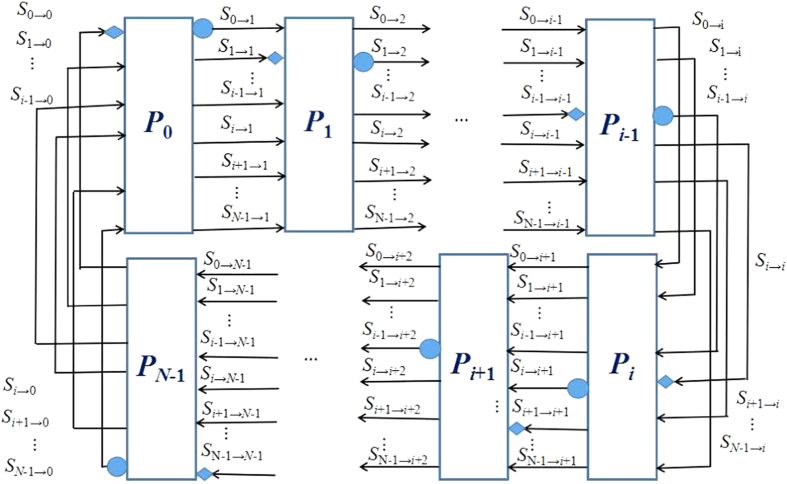
The performance of the proposed MQKA without considering eavesdropping checking. Each participant *P*_*i*_ sends a random two-particle state sequence from the solid circle to the next participant, and with solid diamond as the end. After encoded by all other participants, the sequence is transmitted back to *P*_*i*_.

Suppose that there are *N (N* ≥ 2) participants *P*_0_, *P*_1_, *P*_2_, …, and *P*_*N*−1_, and each of them generate a random sequence with length 2*n* as his or her secret key firstly.


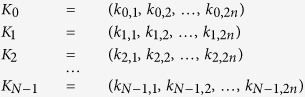


where the element 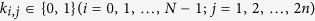. Next, *P*_0_, *P*_1_, *P*_2_, …, and *P*_*N*−1_ want to negotiate a common key 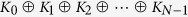. Here, 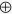 denotes the bitwise Exclusive OR. Now, The detailed description of the proposed MQKA protocol can be seen in Fig. 1 and the following explanation.

#### The Detailed Description of MQKA

##### Step 1 Initialization Phase

Each participant *P*_*i*_ selects two random sequences *S*_*I*_ and *V*_*I*_ with length 2*n*, and prepares a two-particle quantum state sequence *S*_*i,i*+1_ according to the random sequence *S*_*I*_.


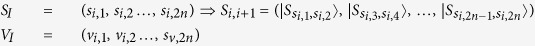


where *s*_*i,j*_, *v*_*i,j*_ ∈ {0, 1} and the definition of 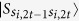 can be seen in equation (1), *i* = 0, 1, …, *N* − 1; *j* = 1, 2, …, 2*n*; *t* = 1, 2, …, *n*.

Next, *P*_*i*_ performs unitary operations 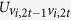 (*t* = 1, 2, …, *n*) on every state 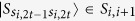, and the resulted sequence be denoted as *S*_*i*→*i*+1_. He also generates *kn (k* is the detection rate) decoy particles from {|0〉, |1〉} or {|+〉, |−〉} randomly, and gets a new sequence 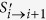 by inserting them into the sequence *S*_*i*→*i*+1_. Meanwhile, *P*_*i*_ records the initial states and corresponding positions of every checking particles, and then sends the sequence 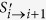 to the next participant *P*_*i*+1_,where + denotes modulo *N* addition.

In addition, it is important to note that the decoy particles could be inserted into *S*_*i*→*i*+1_ randomly. For example, suppose 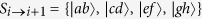 and the decoy sequence is 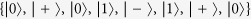 with the position (1, 3, 4, 6, 8, 10, 11, 15), then 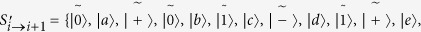

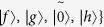
 (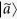 denotes decoy particle). Next, the particles in 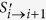 is transmitted one after another.

##### Step 2 Eavesdropping Checking Phase

After confirming that all *P*_*i*+1_ have received the sequence 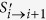, *P*_*i*_ and *P*_*i*+1_ can calculate the error probability by comparing the measurement results with the initial states of decoy particles. If the error ratio exceeds the predetermined threshold value, *P*_*i*_ declares that the communication is invalid. Otherwise, and the process continues to **Step 3**.

##### Step 3 Encoding Phase

By deleting the decoy states from 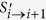, *P*_*i*+1_ can get the sequence *S*_*i*→*i*+1_. Then according to the private key *K*_*i*+1_, *P*_*i*+1_ performs unitary operations 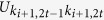 (*t* = 1, 2, …, *n*) on every two-particle state in *S*_*i*→*i*+1_, and denotes the resulted sequence as *S*_*i*→*i*+2_. Here the definition of 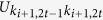 can be seen in equation (2). Next, *P*_*i*+1_ will get a new sequence 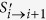 by inserting the decoy particles into *S*_*i*→*i*+2_ similar to **Step 1**, and send it to *P*_*i*+2_.

##### Step 4 Encoding Recursively Phase

After confirming that *P*_*i*+2_ have received the sequence 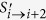, *P*_*i*+1_ and *P*_*i*+2_ execute **eavesdropping checking** mentioned in **Step 2**. If the error ratio exceeds the predetermined threshold value, *P*_*i*_ declares that the communication is invalid. Otherwise, the process continues. *P*_*i*+2_ execute **Encoding Phase** similar to *P*_*i*+1_ in **Step3**.

*P*_*i*+3_, …, *P*_*i*−1_ execute **eavesdropping checking** mentioned in **Step 2** and **Encoding Phase** similar to *P*_*i*+1_ in **Step3**.

##### Step 5 Extracting Common Key Phase

When *P*_*i*_ has received the sequence 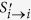 from *P*_*i*−1_, he firstly does eavesdropping checking with *P*_*i*−1_. Then he will obtains the sequence *S*_*i*→*i*_ by deleting the decoy particles from 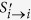. Next, *P*_*i*_ performs unitary operation 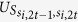 on the corresponding two-particle state in the sequence *S*_*i*→*i*_ according the sequence 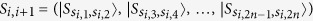, and takes measurements on every resulted two-particle state with basis {00, 01, 10, 11} if *N* is odd, or {+ +, − +, + −, − −} if *N* is even.

- If *N* is odd, denote the sequence of measured result as 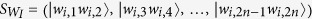. Then *P*_*i*_ computes

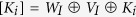

- If *N* is even, denote the sequence of measured result as 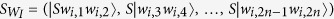. Then *P*_*i*_ computes

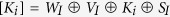


where 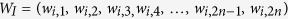.

The 2*n* – *bit* sequence [*K*_*i*_] is the target common key [*K*] of the *N* participants.

#### Correctness of The Proposed Protocol

Now, we show that 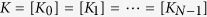.

In fact, the sequence *W*_*I*_ defined in **step 5** can be got by using **Theorem 3** or **Theorem 4** separately. Namely, after performed unitary operations 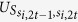 on every two-particle state of sequence *S*_*i*→*i*_, the t-th two-particle state of the resulted sequence can be represented as


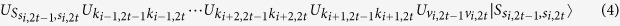


i.e., *P*_*i*_, *P*_*i*+1_, …, and *P*_*i*−1_ perform unitary operations defined by equation (2) on the two-particle state 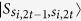 separately, and *P*_*i*_ performs the operation defined by equation (3) at last.

- If *N* is odd, then we can get the conclusion that the *t* – *th* two-particle state mentioned in (4) will be in {|00〉, |01〉, |10〉, |11〉}, and the state of (4) equals 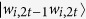 by using **Theorem 3**. Furthermore, we can also get
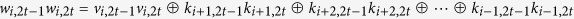
Then,
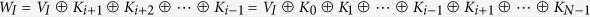
Hence,
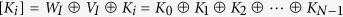
- If *N* is even, then we can get the conclusion that the *t* – *th* two-particle state mentioned in (4) will be in {|+ +〉, |− +〉, |+ −〉, |− −〉}, and the state of (4) equals 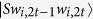 by using **Theorem 4**. Furthermore, we can also get

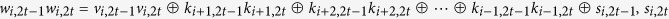


Then,


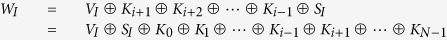


Hence,


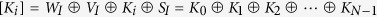


From (i) (ii), we can know that all participants obtain the target common key sequence successfully, i.e.


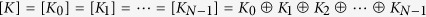


#### An Example of The Proposed Protocol with N = 5

In the following, we will give an example of five-party quantum key agreement protocol without considering eavesdropping checking. Suppose *P*_0_, *P*_1_, *P*_2_, *P*_3_, *and P*_4_ want to negotiate a common sequence with length 6 as the target key. Firstly, they select their private key separately as follows.


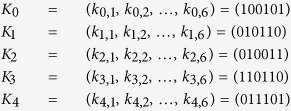


Next,they run the protocol.

##### Step 1 Initialization Phase

*P*_*i*_ selects two random sequences *V*_*I*_ and *S*_*I*_ with length 2*n*, and prepares a two-particle quantum state sequence *S*_*i,i*+1_ according to the random sequence *S*_*I*_.


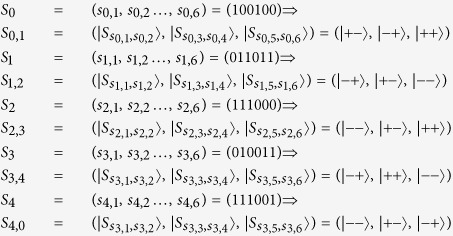



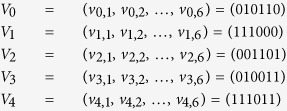


Next, *P*_0_ performs unitary operations 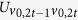 on every state 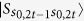 (*t* = 1, 2, 3), and the resulted sequence be denoted as *S*_0→1_. *P*_1_, *P*_2_, *P*_3_ and *P*_4_ perform the same operations similarly. *P*_0_ (or *P*_1_ or *P*_2_ or *P*_3_ or *P*_4_) sends *S*_0→1_ (or *S*_1→2_ or *S*_2→3_ or *S*_3→4_ or *S*_4→0_) to *P*_1_ (or *P*_2_ or *P*_3_ or *P*_4_ or *P*_0_).

##### Step 2 Encoding Phase and Encoding Recursively Phase

*P*_1_ (or *P*_2_ or *P*_3_ or *P*_4_ or *P*_0_) encodes *S*_0→1_ (or *S*_1→2_ or *S*_2→3_ or *S*_3→4_ or *S*_4→0_) by using a unitary operation according to his private key.


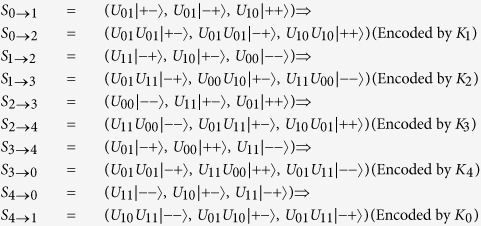


The encoding procession continues until *P*_0_ has received the sequence *S*_0→0_ Encoded by *K*_1_, *K*_2_, *K*_3_, and *K*_4_) separately. *S*_0→0_, *S*_1→1_, *S*_2→2_, *S*_3→3_ and *S*_4→4_ can be represented as follows.


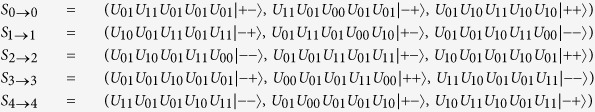


##### Step 3 Extracting Common Key Phase

*P*_0_ (or *P*_1_ or *P*_2_ or *P*_3_ or *P*_4_) performs unitary operations decided by *S*_0,1_ (or *S*_1,2_ or *S*_2,3_ or *S*_3,4_ or *S*_4,0_) on *S*_0→0_ (or *S*_1→1_ or *S*_2→2_ or *S*_3→3_ or *S*_4→4_), and takes measurements on every two-particle state of the resulted sequence with basis {|00〉, |10〉, |01〉, |11〉} because N = 5 is odd. Then the measurement results of *P*_0_ (or *P*_1_ or *P*_2_ or *P*_3_ or *P*_4_) will be


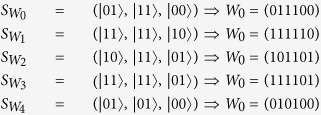


At last, *P*_0_ computes 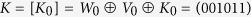, and it is easy to verify that 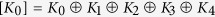. *P*_1_, *P*_2_, *P*_3_ and *P*_4_ can also obtain the target common key sequence 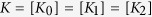 similar to *P*_0_.

#### Security Analysis of The Proposed Protocol

In this section, we will show that the proposed MQKA protocol is secure against external and internal attacks. The external attacks contains intercept-resend attack and entangling attack. Without loss of generality, we only consider the circumstance that there are only three participants named *P*_0_, *P*_1_ and *P*_2_ in the proposed scheme, and it is similar to other cases. Here, we suppose that an eavesdropper named Eve wants to eavesdrop the target common key of *P*_0_, *P*_1_ and *P*_2_ without being detected.

Firstly, let us discuss the intercept-resend attack. Suppose that *P*_0_ prepares a two-particle quantum state sequence *S*_0→1_ according to a random sequence *S*^0^ with length 2*n. P*_0_ inserts 2*n* decoy particles into it and sends the new sequence 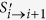 to *P*_1_. If Eve intercepts the sequence and re-sends a fake sequence prepared beforehand instead of 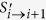, then she wants to obtain the operations performed by *P*_1_ through the fake sequence. However, Eve will be detected with probability 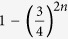 in the eavesdropping check phase by *P*_0_ and *P*_1_ because she does not know about the positions and basis of decoy particles. Hence, Eve will be detected with probability converging to 1 when *n* is large enough. Similar to the intercept-resend attack in the channel between *P*_1_ and *P*_2_ or *P*_2_ and *P*_0_.

Secondly, let us discuss the entangling attack. Suppose Eve intercepts a transmitting particles to the sequence 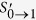, and performs a unitary operation *U*_*e*_ on the intercepted particles to entangle an ancillary particles |*E*〉 prepared beforehand. The unitary operation *U*_*e*_ can be defined by the following equations:


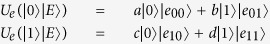


where |*e*_00_〉, |*e*_01_〉, |*e*_10_〉 and |*e*_11_〉 are pure states decided by the unitary operation *U*_*e*_, and the amplitude *a, b, c* and *d* satisfy |*a*|^2^ + |*b*|^2^ = 1 and |*c*|^2^ + |*d*|^2^ = 1. Then it is easy to get:


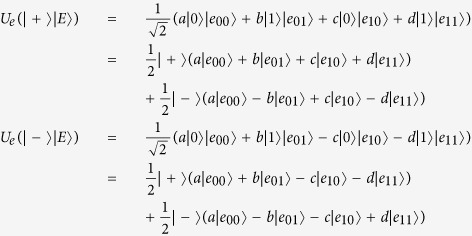


If the decoy particle belongs to {|0〉, |1〉}, in order to pass the eavesdropping checking phase, Eve has to set *b* = *c* = 0 which implies that *a* = *d* = 1. Then Eve cannot distinguish |*e*_00_〉 from |*e*_11_〉, and cannot get any useful information. Hence the entangling attack cannot work in the proposed scheme.

Thirdly, let us discuss the internal attack. Without loss of generality, suppose the dishonest participants, *P*_1_ and *P*_2_, want to cooperate to determine the target common key alone by illegal means. In the encoding procession *P*_0_ → *P*_1_ → *P*_2_ → *P*_0_, *P*_0_ does not leaks any information. In the encoding procession *P*_1_ → *P*_2_ → *P*_0_ → *P*_1_, *P*_0_ encodes the two-particle states by his private key in the last step, and meanwhile, he has already obtained the information of the 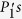 and 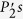 private keys from *S*_0→0_. So we only need to consider the encoding procession *P*_2_ → *P*_0_ → *P*_1_ → *P*_2_. Firstly, *P*_2_ sends *S*2 → 0 to *P*_0_. Meanwhile, he also sends his private information *S*_2_ and *V*_2_ to *P*_1_. Secondly, after the eavesdropping checking phase between *P*_0_ and *P*_1_, *P*_1_ perform unitary operations defined by equation (3) according to the 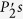 private information *S*_2_. Next, *P*_1_ takes measurements on the two-particle state in the resulted sequence with the basis 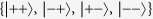. At last, *P*_1_ eavesdrops 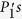 private key successfully from the value of the measurement results, *S*_2_ and *V*_2_. Even so, *P*_1_ and *P*_2_ still can not determine the target common key alone. In fact, it is obvious that the only way to the *P*_0_ to get the target key sequence is to compute 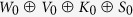, and the information of *V*_0_ and *S*_0_ is only known to *P*_0_. Suppose that *P*_1_ and *P*_2_ embed new private key in the procession *P*_0_ → *P*_1_ → *P*_2_ → *P*_0_, then the behavior of them only affects the value of *W*_0_ because of that *P*_1_ and *P*_2_ know nothing about *V*_0_ and *S*_0_. Therefore, the final key [*K*_0_] of *P*_0_ will be different from the final key [*K*_1_] and [*K*_2_]. Hence, *P*_0_, *P*_1_ and *P*_2_ can not obtain the target common key sequence. In a word, *P*_1_ and *P*_2_ cannot determine the target common key alone by illegal means, and the proposed protocol is secure against internal attack.

#### Efficiency Comparison with Existing Protocol

In this section, we will compare the proposed MQKA protocols with five existing MQKA protocols in the following four aspects: number of qubit measurements, number of unitary operations, qubit efficiency and security against internal attack. The five existing MQKA protocols are “LGHW protocol”^20^, “SZ protocol”^21^, “SZWYZL protocol”^26^, “SYW protocol”^28^, and “SZWLL protocol”^29^. The qubit efficiency can be defined as 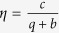, where *c* is the length of target common key sequence, *q* is the number of qubits used in transmission and security checking, and “b” is the number of used classical bits. We only compare the internal attack because the internal attackers are the most powerful attackers in the multi-party protocols usually. Suppose the five protocols just mentioned will produce 2 – *bit* target common key sequence, i.e., *c* = 2. The parameter comparison can be seen in Table 2.

- LGHW protocol. The protocol is secure from internal attack, because it is based on BB84 and all participants transmit their privacy secret only once. However, the efficiency 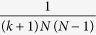 is too low and the number of measurements is larger than others.
- SZ protocol. The efficiency and the number of measurements are both not good. More important, it is susceptible to internal attacks owing to an attack strategy^20^ proposed by Liu, *et al*.
- SZWYZL protocol. Any participant’s modification can be detected by others because the protocol is based on cluster states. Hence, it is secure from internal attack. Besides, I think the efficiency analysed by authors in ref. 26 is not objective. In fact, the efficiency 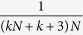 is not good, and the number of measurements and unitary operations are also high.
- SYW protocol. The protocol is similar to SZWYZL protocol, so it is secure for internal attack. The parameters of efficiency, the number of measurements and unitary operations, are all better than SZWYZL protocol.
- SZWLL protocol. The protocol is an improvement on LGHW protocol, and it is much more efficient than any other secure protocols. However, it is susceptible to internal attacks. Without loss of generality, we consider three-party protocol. Suppose the dishonest participants, *P*_1_ and *P*_2_, want to cooperate to obtain the private key of *P*_0_. Consider the message encoding phase in the procession *P*_2_ → *P*_0_ → *P*_1_ → *P*_2_. Firstly, *P*_2_ pre-agreed a common final key [*K*] with *P*_1_, and tells the original state of each photon in the sequence *S*_2_ to *P*_1_. Secondly, after eavesdropping check between *P*_1_ and *P*_0_, *P*_1_ takes measures on 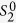 with basis {|0〉, |1〉}, and obtains the privacy *k*_0_ according to *S*_2_. Thirdly, *P*_1_ sends *k*_1_ and *k*_0_ to *P*_2_. At last, *P*_2_ encodes 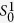 according to 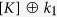. Hence, *P*_0_, *P*_1_ and *P*_2_ obtain the final key [*K*] only determined by *P*_1_ and *P*_2_ only.
- Our protocol. Firstly, our protocol is secure against internal attack. Secondly, The number of measurements is better than LGHW protocol and SZWYZL protocol, but worse than SYW protocol. The unitary operations is not better than LGHW protocol, SZWYZL protocol and SYW protocol. However, the efficiency of our protocol is better than any other secure protocols.

## Discussion

In this paper, we propose the first multiparty QKA protocol based on a quantum search algorithm known as Grover’s algorithm. Firstly, we generalize the properties of quantum search algorithms. Secondly, using the generalized properties of QSA, we propose a multiparty QKA protocol. Next, a 5-party protocol novel example is presented. At last, the security and efficiency analysis shows that our protocol is prior to existing MQKA protocols.

## Additional Information

**How to cite this article:** Cao, H. and Ma, W. Multiparty Quantum Key Agreement Based on Quantum Search Algorithm. *Sci. Rep.* **7**, 45046; doi: 10.1038/srep45046 (2017).

**Publisher's note:** Springer Nature remains neutral with regard to jurisdictional claims in published maps and institutional affiliations.

## Figures and Tables

**Table 1:** The Values of 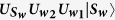 with Different *w, w*_2_ and *w*_1_.

*S*_*w*_/*w*	{*w*_1_, *w*_2_}	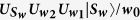
|+ +〉/00	{00, 01} *or* {10, 11}	|− +〉/01
|+ +〉/00	{00, 10} *or* {01, 11}	|+ −〉/10
|+ +〉/00	{00, 11} *or* {10, 01}	|− −〉/11
|+ −〉/10	{00, 01} *or* {10, 11}	|− −〉/11 or −|− −〉/11
|+ −〉/10	{00, 10} *or* {01, 11}	|+ +〉/00 or −|+ +〉/00
|+ −〉/10	{00, 11} *or* {10, 01}	|− +〉/01 or −|− +〉/01
|− +〉/01	{00, 01} *or* {10, 11}	|+ +〉/00 or −|+ +〉/00
|− +〉/01	{00, 10} *or* {01, 11}	|− −〉/11 or −|− −〉/11
|− +〉/01	{00, 11} *or* {10, 01}	|+ −〉/10 or −|+ −〉/10
|− −〉/11	{00, 01} *or* {10, 11}	|+ −〉/10 or −|+ −〉/10
|− −〉/11	{00, 10} *or* {01, 11}	|− +〉/01 or −|− +〉/01
|− −〉/11	{00, 11} *or* {10, 01}	|+ +〉/00 or −|+ +〉/00

**Table 2:** Comparison between the existed five MQKA protocols with our protocol.

*N* – *party* QKA Protocols	*η*	Number of Measurements	Number of Unitary Operations	Security against Internal Attack
LGHW protocol	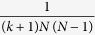	2(*k* + 1)*N(N* − 1)	0	Secure
SZ protocol	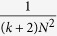	(*k* + 1)*N*^2^	0	Insecure
SZWYZL protocol	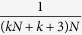	(2*kN* + 2*k* + 3)*N*	*N*^2^	Secure
SYW protocol	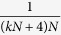	(*kN* + 1)*N*	(*N* − 1)*N*	Secure
SZWLL protocol	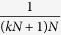	2(*kN* + 1)*N*	(*N* − 1)*N*	Insecure
Our protocol	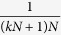	2(*kN* + 1)*N*	(*N* + 1)*N*	Secure
